# NMR reveals the interplay between SilE and SilB model peptides in the context of silver resistance†

**DOI:** 10.1039/d1cc02597j

**Published:** 2021-08-09

**Authors:** Lucille Babel, Minh-Ha Nguyen, Cédric Mittelheisser, Marie Martin, Katharina M. Fromm, Olivier Walker, Maggy Hologne

**Affiliations:** University of Fribourg, Department of Chemistry, chemin du musée 9 Fribourg 1700 Switzerland; Université de Lyon, CNRS, UCB Lyon1, Institut des Sciences Analytiques, UMR 5280, 5 rue de la Doua Villeurbanne 69100 France maggy.hologne@univ-lyon1.fr

## Abstract

SilE and SilB are both proteins involved in the silver efflux pump found in Gram-negative bacteria such as *S. typhimurium*. Using model peptides along with NMR and CD experiments, we show how SilE may store silver ions prior to delivery and we hypothesize for the first time the interplay between SilB and SilE.

## Abstract 2

Illustrative sketch representative of the interplay between the SilB and SilE proteins in the context of silver bacteria resistance. In case of an excess of silver, SilE plays the role of a regulator and avoids a collapse of the silver efflux pump.

## Graphical abstract

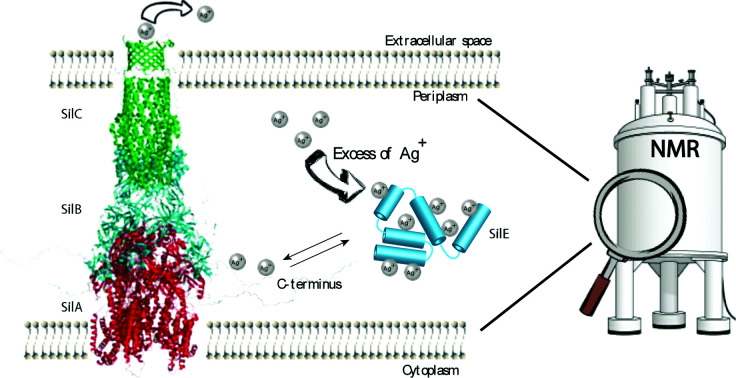

The antimicrobial properties of silver have extensively been used for thousands of years. We currently find silver in many every day used devices like silver based wound dressings, cosmetics, sanitary towels or shower gels. Despite this long-standing history and its demonstrated activity against Gram-negative bacteria, the complete bactericidal mode of action of silver at a molecular level remains unclear. To counteract the toxic effect of silver, some Gram-negative bacteria have developed different resistance mechanisms, including the efficient transport of the metal ions out of the cell promoted by an efflux pump. The plasmid pMG101 which was isolated from *Salmonella* strains after the death of patients in the burn ward at the Massachusetts General Hospital is the best characterized silver-resistance system.^1^ The silver-resistant gene cluster is composed of nine genes: a chemiosmotic Ag^+^/H^+^ efflux pump (SilCBA), an ATPase efflux pump (SilP), a responder and membrane sensor performing two-component transcription regulation (SilRS) and two periplasmic silver-binding proteins SilE and SilF.^2^ SilE is an interesting target to understand the silver resistance since this protein is only synthesized during bacterial growth in the presence of silver ions. Following the current literature, SilE is predicted as an intrinsically disordered protein (IDP) in the free state and folds into helices in the presence of silver.^3^ Due to the fact that SilE can bind several silver ions, it has been qualified as a “molecular sponge”.^4^ However, no clear evidence nor experimental data has allowed to characterize its exact folding and silver-binding mechanism. In particular, high-solution structural data on the free disordered SilE and the Ag–SilE complex are lacking to grasp the coordination mechanisms at a molecular level. Recently, a significant step forward has been achieved with the refinement of Ag–SilE derived peptide structures and their binding affinities.^5,6^ This allowed to emphasize that histidine and methionine residues are involved in the silver binding event leading to the helical folding of the peptides. Beyond structure, essential questions concerning the role of SilE in the efflux pump and its functional role with respect to other proteins of the system still constitutes an unresolved conundrum. It is thus tempting to resort to a homologous system to answer these dangling questions. Indeed, the silver efflux pump is very similar to the copper resistance pump with the presence of the CusCFBA transporter. This transporter is a closely related homologue to the SilCFBA transporter with most of the counterpart proteins showing amino acid sequence identity of *ca.* 80%.^7^ The structure of CusCBA has been extensively characterized^8–10^ where it undergoes a stepwise process and uses a network of methionine entities to transport silver or copper ions out of the cell. Furthermore, metal ion delivery to the CusCBA transporter has been ascribed to CusF that could transfer an ion to the long N-terminal tail of CusB containing a cluster of three methionine residues.^11–13^ Using the Cus system to devise the mechanistic function of Sil may be of interest but also limited for several reasons. First and foremost, the SilB and CusB show a low degree of identity in both the N and C-termini (see Fig. S1, ESI†) where the C-terminus of SilB comprises 15 more amino acids and three extra methionine residues compared to its counterpart in CusB. Although a previous work on SilB from *Cupriavidus metallidurans* CH34 has concluded to a metal transfer from the N to the C-terminus, this conclusion does not apply to the SilB from *Salmonella typhimurium* due to the absence of the Cus-F like extra residues where the interaction is delineated (see Fig. S2, ESI†).^14^ Second, the SilE protein present in the Sil system has no counterpart in the Cus system. SilE is the only other Sil system component aside from SilCFBA that is essential for the exogenous silver resistance phenotype and it has been postulated that SilE does not only act as a molecular sponge by sequestering silver ions but also acts in an analogous manner to SilF/CusF and chaperones silver to SilCBA for efflux, either directly or *via* SilF.^7^ At this level, it is likely that SilF would interact with the N-terminus of SilB if we consider that the Sil system adopts a similar mechanism compared to the Cus system. A step forward is to elucidate the role played by SilE, not present in the Cus system, in the mechanism of silver transfer (if any) to SilABC. More precisely, it is mandatory to comprehend how SilE may possibly interact with SilABC to release silver when SilE is silver-saturated. Since SilABC is organized as a multimeric architecture^10^ and only SilB has been shown to interact with periplasmic proteins to activate the metal pump,^15^ we have chosen to focus our study on SilB on one side. Moreover, the C-terminus of SilB that has no homologs in the Cus system, appears as a plausible interaction site (see Fig. 1 and ESI†) and has encouraged us to engineer the SilB^401-430^ peptide (denoted hereafter SilB-p). We have hypothesized that this structural part may be involved in a regulation mechanism with SilE where histidine and methionine residues have been shown to represent the main interaction residues with silver.^6^ This, added to the fact that the following mutations H80A, H87A, M83L or M90L induce a large folding defect of SilE upon silver binding,^16^ has incited us to engineer and study the SilE^80-90^ peptide (denoted hereafter SilE-p and seen in Fig. 1 and ESI†).

**Fig. 1:**
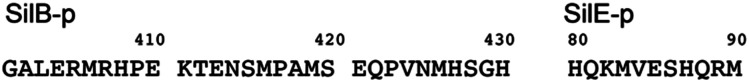
The sequence of SilB-p is extracted from the complete sequence of SilB *Salmonella typhimurium* (Uniprot Q9ZHD0, see ESI†). The sequence of SilE-p is extracted from the complete sequence of SilE *Salmonella typhimurium* (Uniprot Q9Z4N3, see ESI†).

We report qualitative and quantitative aspects of the interplay between SilE-p and SilB-p with respect to silver binding and transfer. We have first investigated the ability of SilE-p to interact with SilB-p by means of NMR. To this end, we have assigned the corresponding NMR chemical shifts of SilE-p and SilB-p by a combination of ^1^H–^1^H TOCSY and ^1^H–^1^H NOESY experiments (see ESI†). The corresponding ^1^H,^15^N-HSQC spectra for SilE-p and SilB-p exhibit an ensemble of chemical shifts distributed on a narrow region in the ^1^H frequency dimension (Fig. S3A and B and ESI† for assignments). This hallmark, added to the fact that no NOE between amide protons of residues *i*, *i* + 3 could be detected, is a clear indication that SilE-p and SilB-p are disordered in the free state.^17^ To detect a potential interaction between SilE-p and SilB-p, we monitored signal shifts in a ^1^H,^15^N-HSQC spectrum of a SilE-p/SilB-p mixture at a 1 : 1 molar ratio and compared it with their isolated ^1^H,^15^N-HSQC spectra in the free state. As shown on Fig. S3C and D (ESI†), the spectra of the isolated SilE-p and SilB-p nicely overlap with the spectrum corresponding to the SilE-p/SilB-p mixture and therefore provide a clear evidence that SilE-p and SilB-p do not interact with each other. To map the interaction of silver with either SilE-p or SilB-p, we monitored the chemical shift perturbations (CSPs) in a series of ^1^H,^15^N-HSQC spectra upon addition of silver ions. As illustrated on Fig. 2, several residues on both SilE-p or SilB-p present significant CSPs while some showed a strong decrease of their signal intensities, indicative of intermediate exchange. It has to be recalled that CSPs include either, even light, structural rearrangement and interaction with silver ions. On SilE-p, the most affected residues cluster around two binding sites that comprise K82, M83 and R89, M90 (see Fig. 2A). Additionally, M83 and M90 exhibit a strong signal broadening indicative of intermediate-to-slow exchange and hence a tight binding with silver ions. The magnitude of the CSPs of residues participating in interaction saturate at approximately 2 : 1 molar ratio of silver ions to SilE-p and therefore indicates a 2 : 1 stoichiometry, in good agreement with the proposed presence of two binding sites (see Fig. 3A). To quantify the binding interactions, we have used a 2 : 1 binding model (see ESI†) and assumed two binding sites with identical affinity. We have derived a dissociation constant of 4.5 ± 1.5 μM from the NMR titration data in good agreement with the *K*_d_ reported previously *via* a different method.^6^ By means of the same methodology, we have investigated the interaction of SilB-p with silver ions. As can be seen on Fig. 2B, the most significant CSPs are located around R407 flanked by M406 and H408 while a second binding site is identified around A418, involving M416 and M419. Surprisingly, M426 does not show any significant CSP_S_, thus does not participate in silver binding. A closer inspection of the corresponding CSPs of the two binding sites reveals a distinct behaviour upon silver titration. The first site that induces chemical shifts of M416, A418 and M419 exhibits a hyperbolic titration curve that reaches a plateau after 2 equivalents of silver ions (see Fig. 3B). In the case of the second binding site resulting in CSPs of R405, M406, R407 and H408, the titration curve shows a sigmoidal shape and suggests a cooperative silver binding. Moreover, the Ag^+^ concentration needed to reach the bond state is twice compared to the previous binding site. This effect has already been underlined in several other cases where intrinsic disorder might optimize allosteric coupling in proteins.^18^ To quantify the binding affinity of silver with SilB-p, we have used a sequential model that takes into account the cooperative binding of the silver ions. This model is based on two binding events and thus relies on two dissociation constants associated with the two binding sites (see Appendix 1, ESI†). Overall, we derived two *K*_ds_ of 4 ± 2 and 571 ± 80 μM respectively, showing a clear difference between the two SilB-p binding sites and a negative cooperativity of the second binding site. The lowest *K*_d_ is associated with the site that primarily saturates and encompasses M416–M419 while the highest *K*_d_ is committed to the binding site that saturates at higher [Ag^+^] concentration and encompasses R405-H408. To rule out the possibility that the much higher *K*_d_ of the second binding site could result from its own affinity, we have engineered two other shorter peptides SilB-p1 and SilB-p2 that comprise the two individual binding sites (see ESI†). Mass spectrometry reveals that each shorter peptide is prone to bind one silver ion (see Fig. S4, S5 and Table S1, ESI†). Additionally, as can be seen on Table S2 and Fig. S6, S7 (ESI†), the derived *K*_ds_ for SilB-p1 and -p2 have a similar affinity with a *K*_d_ of 8 ± 2 and 2 ± 1 μM respectively. It therefore supports a negative cooperative binding effect solely detected in the case of SilB-p that comprises the two binding sites.

**Fig. 2:**
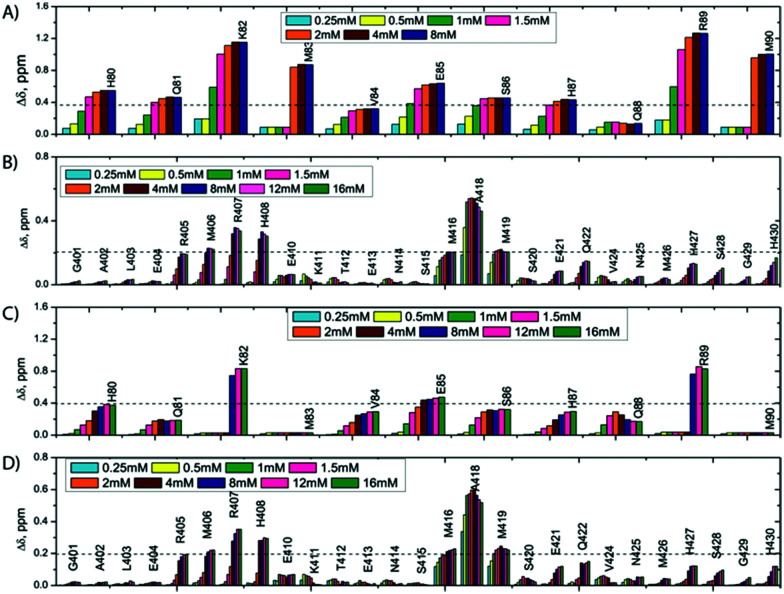
CSPs observed along Ag^+^ titration on a 1 mM SilE-p (A), 0.92 mM SilB-p (B), SilE-p starting from a preformed 0.88 mM SilE-p/SilB-p sample (C), SilB-p starting from a preformed 0.88 mM SilE-p/SilB-p sample (D).

**Fig. 3:**
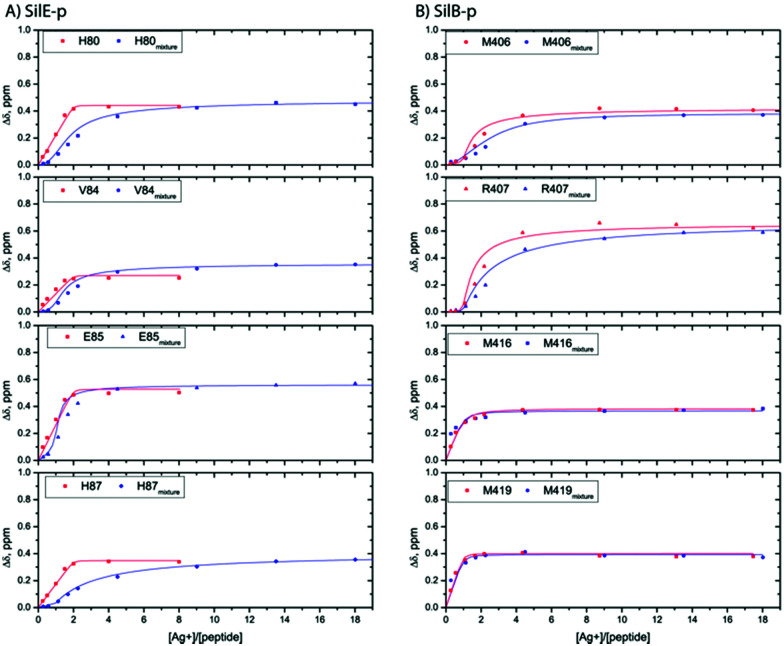
Representative titration curves for SilE-p starting from a free state of SilE-p or a mixture of SilE-p/SilB-p as a function of the molar ratio of Ag^+^ (A) and SilB-p starting from a free state of SilB-p or a mixture of SilB-p/SilE-p as a function of the molar ratio of Ag^+^ (B). Symbols represent experimental data while plain lines represent fitted data according to the models presented in ESI.†

A possible explanation for the decrease in affinity may be due to an increase of energy required for the second binding site to reach the bond state. According to these observations, it is likely that silver ions first bind to the site that embraces M416 and M419 and then to the second site including M406. This clearly pinpoints a new binding site for SilB that has neither been detected in its CusB^13^ counterpart nor in SilB from *Cupriavidus metallidurans*.^14^ Conversely to SilE-p that undergoes structural folding upon silver binding, SilB-p remains fully unstructured in its silver-bound state. This finding is supported by several observations. First and foremost, we did not detect any NOE contact between amide protons of residues *i*, *i* + 3 in the silver-bound SilB-p. Second, we have observed low ellipticity above 210 nm and negative bands near 195 nm during a silver titration in circular dichroism experiments (see Fig. S9, ESI†).

That SilE-p and SilB-p both bind silver ions has encouraged us to investigate their interplay. Accordingly, we used an equimolar mixing of SilE-p and SilB-p to which we added a graduated amount of silver ions from [Ag^+^] : [peptide] ratios of 1 : 1 to 8 : 1 with respect to SilE-p or SilB-p. From a first glimpse at Fig. 2C and D, SilE-p and SilB-p present similar binding sites for silver whether they are taken individually or together. Nevertheless, their CSP intensities or titration curves present several discrepancies.

From the SilB-p side, the measured CSPs show a similar pattern compared to those recorded for an independent SilB-p alone in the presence of silver (compare with Fig. 2B). Moreover, the titration curves corresponding to SilB-p or the SilB-p/SilE-p mixture nicely overlap upon addition of silver (Fig. 3B) for the first binding site that encompasses M416 and M419. As a result, our data support the fact that the presence of SilE-p does not affect the binding of silver on the first site of SilB-p. For the second binding site that clusters around M406, only a slight difference could be detected in the titration curves with a slower increase of the saturation rate. The picture is drastically different for the SilE-p in such a mixture as (i) the recorded CSPs display weaker values at saturation for both binding sites (Fig. 2D) and (ii) the titration curves present different shapes that become sigmoidal. Additionally, a larger amount of silver is necessary for SilE-p to reach a plateau (see Fig. 3A). These experimental results strongly support the fact that when SilE-p and SilB-p are both present, SilB-p first binds silver before SilE-p could accommodate the remaining silver ions.

To lend credence to our hypothesis, we carried out competition assays. Starting from a preformed SilB-p/Ag^+^ complex at a [SilB-p]/[Ag^+^] ratio of 1 : 2, we added SilE-p at a [SilB-p]/[SilE-p] ratio of 1 : 1. According to Fig. 4B, the SilB-p binding site that encompasses M419 does not display any signal shift. Conversely, the addition of SilE-p caused the second binding site that clusters around M406 to shift toward the original position they occupied in the free state. We then increased the amount of Ag^+^ to reach a [SilB-p]/[Ag^+^] ratio of 1 : 4 and finally 1 : 8 (Fig. 4C and D). While the first binding site (M419) does not experience any further shift, the signals associated with the second binding site (R405) shift back to the position they occupied at a 1 : 2 ratio and reach a plateau at a 1 : 8 ratio. Our observations clearly demonstrate that SilE-p outcompetes the second binding site of SilB-p for silver binding and acts as a regulator when the silver concentration increases. This mechanism allows SilB-p to bind silver at a much higher concentration compared to its concentration if SilE-p would be absent.

**Fig. 4:**
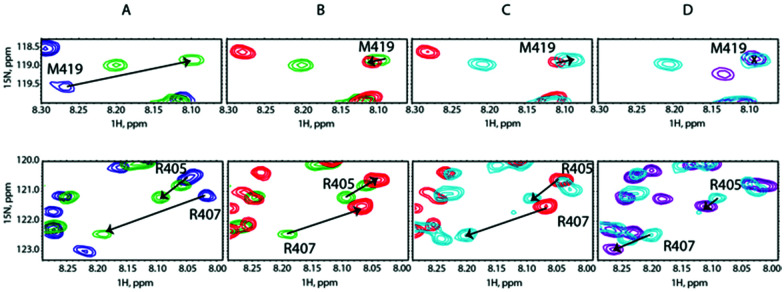
Overlay of representative regions of ^1^H,^15^N-HSQC NMR spectra of (A) SilB-p free in solution (blue contours), after adding Ag^+^ at a [SilB-p]/[Ag^+^] molar ratio of 1 : 2 (green contours), (B) after adding SilE-p at a [SilB-p]/[SilE-p] molar ratio of 1 : 1 (red contours), (C) after adding Ag^+^ at a [SilB-p]/[Ag^+^] molar ratio of 1 : 4 (light blue contours) and (D) after adding Ag^+^ at a [SilB-p]/[Ag^+^] molar ratio of 1 : 8 (purple contours).

We carried out a similar experiment from the SilE-p side with a preformed SilE-p/Ag^+^ complex at a [SilE-p]/[Ag^+^] ratio of 1 : 2 (Fig. 5A). We then added SilB-p at a [SilB-p]/[SilE-p] ratio of 1 : 1 that causes the signals associated with both binding sites to shift back to the position they occupied in the free state of SilE-p.

**Fig. 5:**
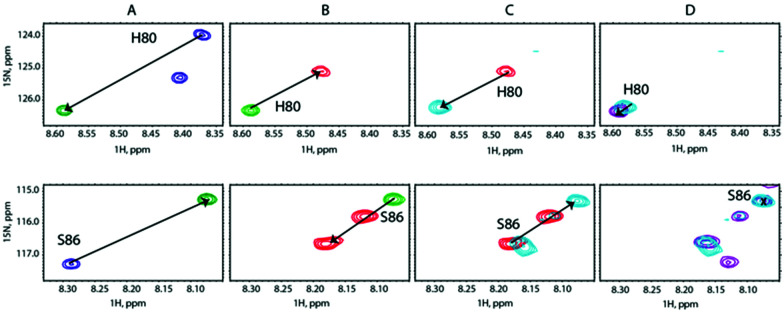
Overlay of representative regions of ^1^H,^15^N-HSQC NMR spectra of (A) SilE-p free in solution (blue contours), after adding Ag^+^ at a [SilE-p]/[Ag^+^] molar ratio of 1 : 2 (green contours), (B) after adding SilB-p at a [SilB-p]/[SilE-p] molar ratio of 1 : 1 (red contours), (C) after adding Ag^+^ at a [SilE-p]/[Ag^+^] molar ratio of 1 : 4 (light blue contours) and (D) after adding Ag^+^ at a [SilE-p]/[Ag^+^] molar ratio of 1 : 8 (purple contours).

Nevertheless, one can notice that this effect is less pronounced than the one experienced by the second binding site of SilB-p when SilE-p is added. This is likely due to the presence of the first and strong binding site of SilB-p that outcompetes SilE-p. Adding a further amount of silver causes the signals to saturate at a 1 : 4 ratio with no further variation at 1 : 8 (Fig. 5C and D). These competition assays led us to hypothesize that SilE-p may bind the excess of silver that SilB-p would not accommodate when the silver concentration increases.

Based on this set of experimental observations, we may draw a conclusion related to the interplay mechanism between SilB-p and SilE-p. While the function or silver binding capability of the C-terminus of SilB from *Salmonella typhimurium* has never been reported, neither in the case of its counterpart in the Cus system nor in the case of SilB from *Cupriavidus metallidurans*, we have shown that either SilB-p or SilE-p possess two silver binding sites with different affinities. In the case of SilE-p, the two binding sites present a similar affinity in the micromolar range while SilB-p exhibits two binding sites that show a negative cooperativity upon silver binding. From our findings, we can hypothesize a synergetic mechanism between SilE and SilB. The negative binding cooperativity of SilB is likely to obey to a rapid remodelling of the system after a significant modification of the silver concentration. We can hypothesize that the C-terminus of SilB would slowly usher silver ions from M406 to M419 prior to a possible release to SilC *via* SilA. When the silver concentration significantly increases, SilE may play the role of a regulator that stores silver ions to avoid an overload of SilB. With such a mechanism, there is no disruption of the silver withdrawal from the cell and therefore maintenance of the silver resistance (see ESI† for a sketch of the possible interplay between SilB and SilE). Of course, this assumption has to be demonstrated in the context of the full native proteins SilE and SilB in a further study.

K. M. F. had the idea to study SilE and excerpts thereof and its possibility to act as chaperone to transfer silver ions to SilB (SNSF project 178827) and corrected the final manuscript. L. B. carried out mass spectrometry and 1D NMR studies, C. M. synthesized the SilB peptides, M. H. N. and M. M. carried out 2D NMR spectroscopy and CD experiments, M. H. carried out 2D NMR experiments and analysis. Finally, M. H. and O. W. wrote the manuscript. This project has also received financial support from the CNRS through the MITI interdisciplinary programs.

## Conflicts of interest

There are no conflicts to declare.

## Supplementary Material

CC-057-D1CC02597J-s001
