# Supplementary material for: NMR reveals the interplay between SilE and SilB model peptides in the context of silver resistance
Source: Chem Commun (Camb). 2021 Aug 9;57(70):8726–9. doi: 10.1039/d1cc02597j (PMC8686694; doi:10.1039/d1cc02597j)
Supplement: CC-057-D1CC02597J-s001 [file CC-057-D1CC02597J-s001.pdf]

Electronic Supplementary Material (ESI) for ChemComm.
This journal is © The Royal Society of Chemistry 2021

Supporting information

NMR reveals the interplay between SilE and SilB model peptides in the context of silver resistance.

Lucille Babel, Minh-Ha Nguyen, Cédric Mittelheisser, Marie Martin, Katharina M. Fromm, Olivier Walker and Maggy Hologne

Peptide sequences. The sequence of SilB-p is extracted from the complete sequence of SilB *Salmonella typhimurium* (Uniprot accession number Q9ZHD0) and is displayed below in yellow.

```
        10         20         30         40         50
MASLKIKYAA IIISSLIAGG LISVTAWQYL NSSQKTVPAE QKAPEKKVLF
        60         70         80         90        100
WYDPMKPDTK FDKPGKSPFM DMDLVPKYAD ESGDKSSGGI RIDPTQVQNL
       110        120        130        140        150
GLKTQKVTRG MLNYSQTIPA NVSYNEYQFV IVQARSDGFV EKVYPLTIGD
       160        170        180        190        200
HVKKGTPLID ITIPEWVEAQ SEFLLLSGTG GTPTQIKGVL ERLRLAGMPE
       210        220        230        240        250
EDIQRLRSTR TIQTRFTIKA PIDGVITAFD LRTGMNISKD KVVAQIQGMD
       260        270        280        290        300
PVWISAAVPE SIAYLLKDTS QFEISVPAYP DKTFHVEKWN ILPSVDQTTR
       310        320        330        340        350
TLQVRLQVTN KDEFLKPGMN AYLKLNTQSQ EMLLIPSQAV IDTGKEQRVI
       360        370        380        390        400
TVDDEGKFVP KQIHVLHESQ QQSGIGSGLN EGDTVVVSGL FLIDSEANIT
       410        420        430
GALERMRHPE KTENSMPAMS EQPVNMHSGH
```

Smaller peptides of SilB-p were also studied such as Ac-GALERMRHPEK-NH_2 and Ac-EKTENSMPAMSE-NH_2 hereafter designated by SilB-p1 and SilB-p2.

The sequence of SilE-p is extracted from the complete sequence of SilE *Salmonella typhimurium* (Uniprot accession number Q9Z4N3) and is displayed below in blue.

```
        30         40         50         60
TETVNIHERV NNAQAPAHQM QSAAAPVGIQ GTAPRMAGMD
        70         80         90        100
QHEQAIIAHE TMTNGSADAH QKMVESHQRM MGSQTVSPTG
       110        120        130        140
PSKSLAAMNE HERAAVAHEF MNNGQSGPHQ AMAEAHRRML

SAG
```

NMR experiments. 2D NMR experiments were acquired at 293 K on a 600 MHz Bruker Avance III HD spectrometer equipped with a triple-resonance probe. The NMR samples (1 mM) have been prepared in 10 mM ammonium acetate at pH 6.4. A capillary containing the lock solvent (D_2O) has been inserted into the tube to avoid any H-D exchange between D_2O and the amide protons of the peptides. NMR assignments of the two peptides have been carried out using 2D ^1H-^1H homonuclear TOCSY (mixing time 80 ms) and NOESY (mixing time 400 ms) experiments. The interaction between peptides have been characterized using chemical

shift perturbation (CSP), where a series of ^1H-^{15}N HSQC spectra have been recorded for each peptide while adding small volumes of silver stock solution (390 mM $AgNO_3$). No labelled peptides have been used for this study and NMR spectra were acquired at ^{15}N natural abundance. To counteract the low abundancy of nitrogen, sofast ^1H-^{15}N HSQC with 140 scans per spectra were recorded. The combination of homonuclear and heteronuclear experiments recorded at different concentrations of silver allowed us to assign completely ^1H-^{15}N spectra of each peptide. Those spectra are used as fingerprint of the two peptides for the interaction studies.

CD experiments. CD experiments were acquired at 293 K on a Chirascan spectrometer (Applied Photophysics). Peptide concentration was 10 µM. Each sample was prepared in 10 mM acetate ammonium solution at pH 6.4. Five repetitions have been recorded for each ratio peptide / Ag^+.

Solid-Phase Peptide Synthesis (SPPS). Peptides were synthesized by SPPS (Solid-Phase Peptide Synthesis) either on a ChemMatrix® Rink-Amide resin (Biotage) for C-terminated amides (SilE-p, SilB-p1 and SilB-p2) on a 108 µmol scale with a Biotage Initiator+ Alstra automated peptide synthesizer. The resin has been swelled in DCM (dichloromethane) during 60 min. 9-fluoromethoxy-carbonyl (Fmoc)-protected amino acids (Bachem) were coupled by using HCTU (O-(1H-6-Chlorobenzotriazole-1-yl)-1,1,3,3- tetramethyluronium hexafluorophosphate, Novabiochem) and HOBt (Hydroxybenzotriazole, Sigma-Aldrich) as coupling agents, DIPEA (N,N-diisopropylethylamine, Sigma-Aldrich) as organic base, and DMF (N,N′-dimethylformamide) and NMP (N-Methyl-2-Pyrrolidone) as solvents, during 60 min. After each coupling, a mixture of acetic anhydride (Acros Organics)/pyridine (Acros Organics)/DMF (1:2:7) has been added to the resin during 10 min in order to protect the unreacted functional groups. Fmoc deprotection steps were carried out twice (3 min and 10 min) by using 20% piperidine (Sigma-Aldrich) in DMF. The N-terminus was acetylated by using a mixture of acetic anhydride/pyridine/DMF (1:2:7) during 10 min. After each step, the solvent has been removed by filtration and the resin has been washed four times with DMF. Six washes with DCM have been performed after the final capping step. Side chain deprotection and peptide cleavage from the resin were carried out by adding 8 mL of a cocktail of 95.5 vol% TFA (trifluoroacetic acid, Sigma-Aldrich), 1.5 vol% EDT (ethane dithiol, Sigma-Aldrich), 1.5 vol% TIS (triisopropylsilane, Sigma-Aldrich) and 1.5 vol% water during 2 h. The TFA has been evaporated under vacuum and the peptides were precipitated and washed 3 times with cold diethyl ether. Peptides were dried and purified by semi-preparative reverse-phase HPLC (Waters 600) on a NUCLEODUR C18 HTec Column (Macherey-Nagel) with a linear gradient from 10% to 50% acetonitrile in water with 0.1% TFA, and then lyophilized. Characterization of the peptides was performed by ESI-MS (Bruker Esquire HCT) and the purity (> 95%) was controlled by analytical HPLC (Waters alliance).
Part of the 2D NMR studies were performed with a batch purchased from "Genosphere".

Mass spectrometry. Peptides were diluted (100-500 µM) in 10 mM solution of ammonium acetate at pH 7.0 and 7.8 and silver nitrate was added (0-4 eq.). Mass spectra at pH 6.4 were also run for consistency and the same complexes were observed. Due to the low signals of the complexes compared to protonation, no spectra at this pH are shown.

Sequence alignment. The alignment has been performed by PRALINE[1-2]. The scoring scheme works from 0 for the least conserved alignment position, up to 10 for the most conserved

alignment position. The peptide sequence SilB$^{401\text{-}430}$ has been displayed using a purple rectangle on each alignment.

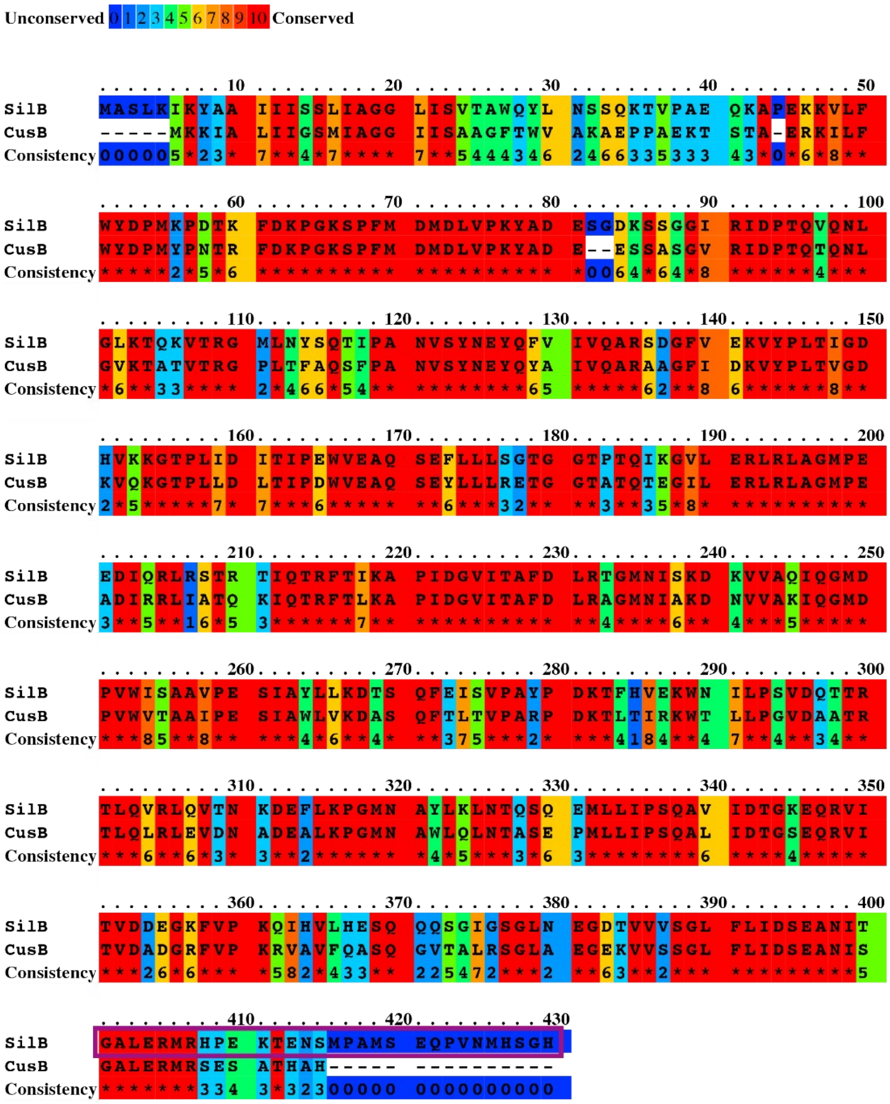

Figure S1. Sequences alignment for SilB *Salmonella typhimurium* / CusB *Escherichia coli K12.* The sequence of the SilB model peptide used in this study has been framed in purple.

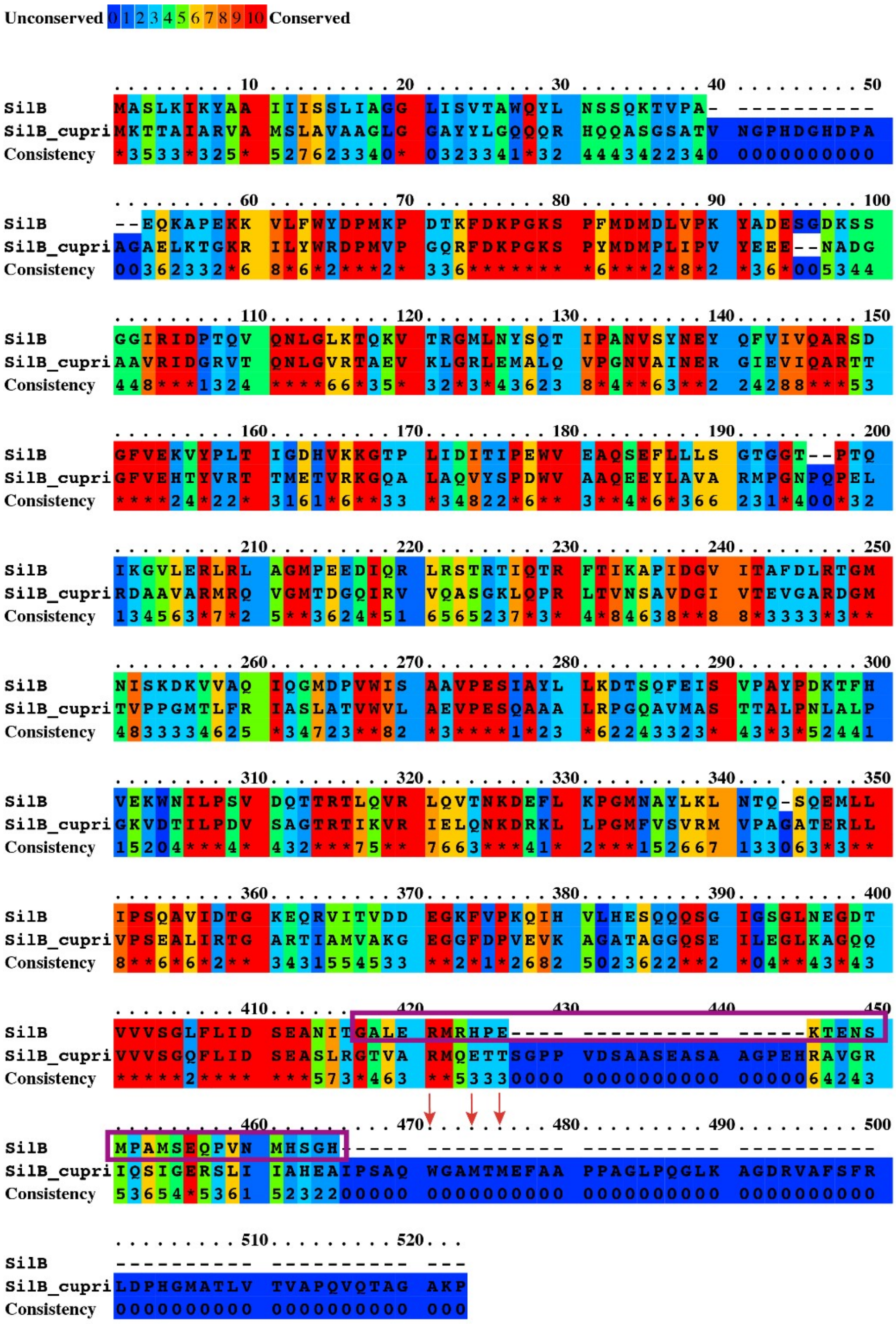

Figure S2. Sequences alignment for SilB *Salmonella typhimurium* / SilB *Cupriavidus metallidurans* CH34. The sequence of the SilB model peptide used in this study has been framed in purple. Red arrows represent the Ag^+ interacting residues seen in the C-terminus of SilB *Cupriavidus metallidurans* CH34.

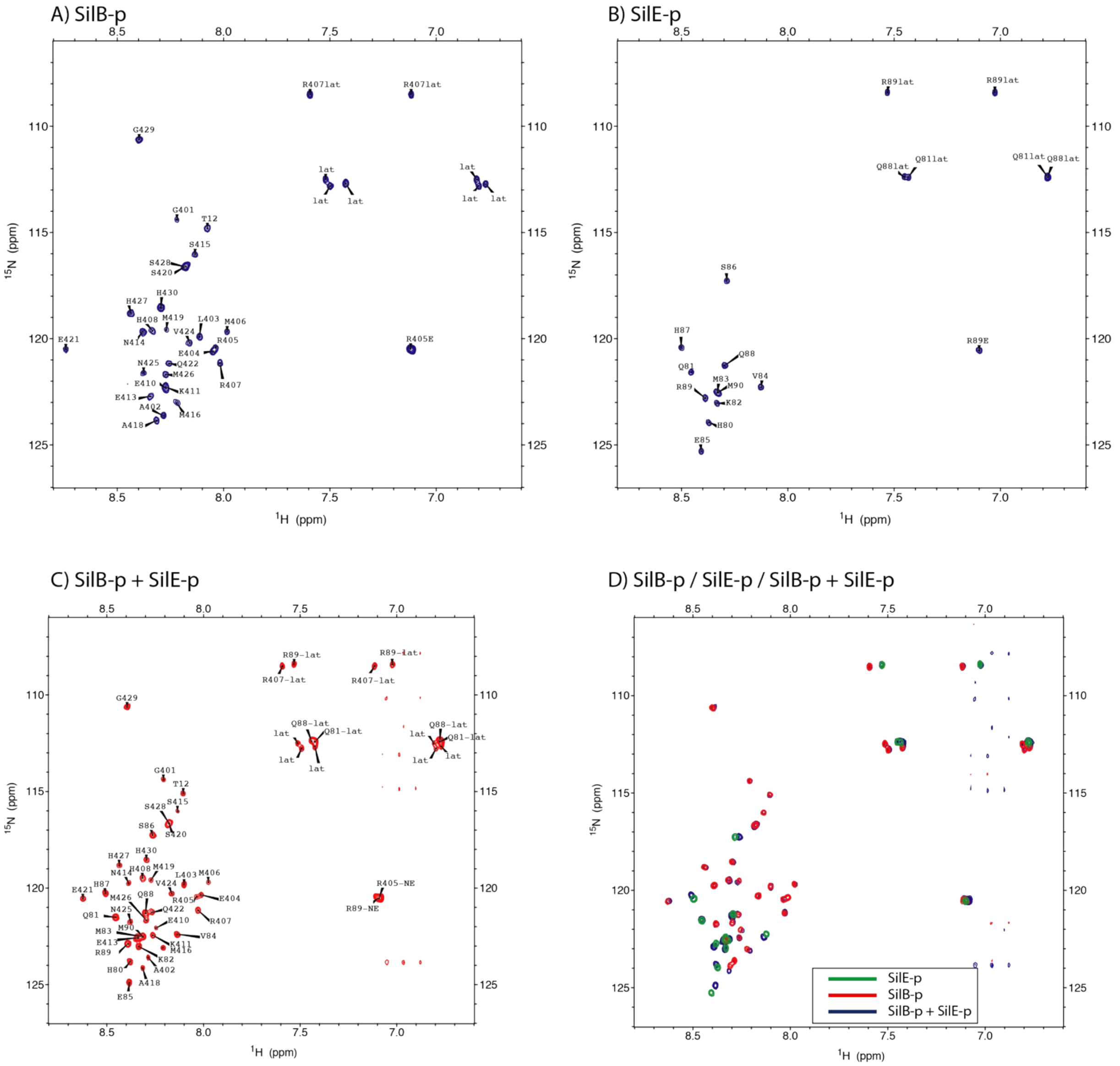

Figure S3. ^{1}H-^{15}N HSQC spectra and respective assignments of the different peptides (A) SilB-p, (B) SilE-p and (C) a mixture of SilE-p and SilB-p at a [SilE-p]/[SilB-p] molar ratio of 1:1. (D) Overlay of the SilE-p (green), SilB-p (red) and SilB-p/SilB-p (blue) ^{1}H,^{15}N-HSQC spectra. The three spectra nicely overlap and evidence the fact that SilE-p and SilB-p do not interact with each other.

^{1}H-^{15}N HSQC chemical shift assignments

1. SilE-p

Amino-acid	Atom	^{1}H, ppm	Atom	^{15}N, ppm
H80	H_N	8.373	N	123.965
Q81	H_N	8.454	N	121.573
	H_{E21}	7.433	N_{E2}	112.497
	H_{E22}	6.777		
K82	H_N	8.330	N	122.98
M83	H_N	8.328	N	122.497
V84	H_N	8.123	N	122.245
E85	H_N	8.406	N	125.258
S86	H_N	8.283	N	117.252
H87	H_N	8.497	N	120.438
Q88	H_N	8.294	N	121.222
	H_{E21}	7.45	N_{E2}	112.378
	H_{E22}	6.777		
R89	H_N	8.382	N	122.729
	$H_{1*/2*}$	7.529	$N_{H1/2}$	108.401
	$H_{2*/1*}$	7.027	$N_{H2/1}$	108.388
	H_E	7.100	N_E	120.542
M90	H_N	8.328	N	122.497

2. SilB-p

Amino-acid	Atom	^{1}H, ppm	Atom	^{15}N, ppm
G401	H_N	8.209	N	114.385
A402	H_N	8.288	N	123.613
L403	H_N	8.101	N	119.801
E404	H_N	8.013	N	120.367
R405	H_N	8.034	N	120.473
	H_E	7.105	N_E	120.489
M406	H_N	7.979	N	119.686
R407	H_N	8.030	N	121.172
	$H_{1*/2*}$	7.594	$N_{H1/2}$	108.511
	$H_{2*/1*}$	7.118	$N_{H2/1}$	108.497
H408	H_N	8.319	N	119.478
E410	H_N	8.256	N	122.020
K411	H_N	8.261	N	122.433
T412	H_N	8.105	N	115.087
E413	H_N	8.335	N	122.559
N414	H_N	8.39	N	129.751
S415	H_N	8.137	N	116.06
M416	H_N	8.223	N	123.019
A418	H_N	8.309	N	123.872
M419	H_N	8.263	N	119.519
S420	H_N	8.180	N	116.653
E421	H_N	8.628	N	120.563
Q422	H_N	8.267	N	121.247
V424	H_N	8.165	N	120.281
N425	H_N	8.382	N	121.729
M426	H_N	8.298	N	121.662
H427	H_N	8.441	N	118.798
S428	H_N	8.180	N	116.653
G429	H_N	8.398	N	110.631
H430	H_N	8.299	N	118.534

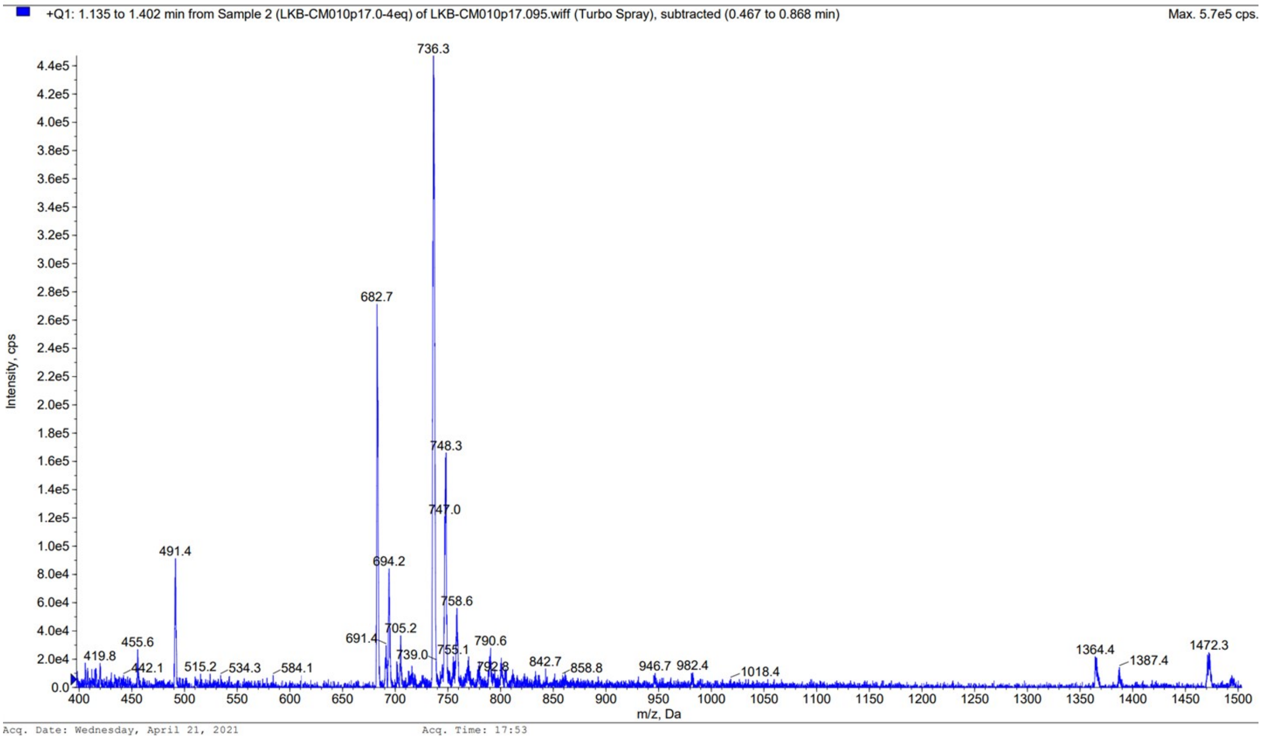

Figure S4. Mass spectrum of SilB-p1 in presence of 4 equivalents of silver nitrate at pH 7.0.

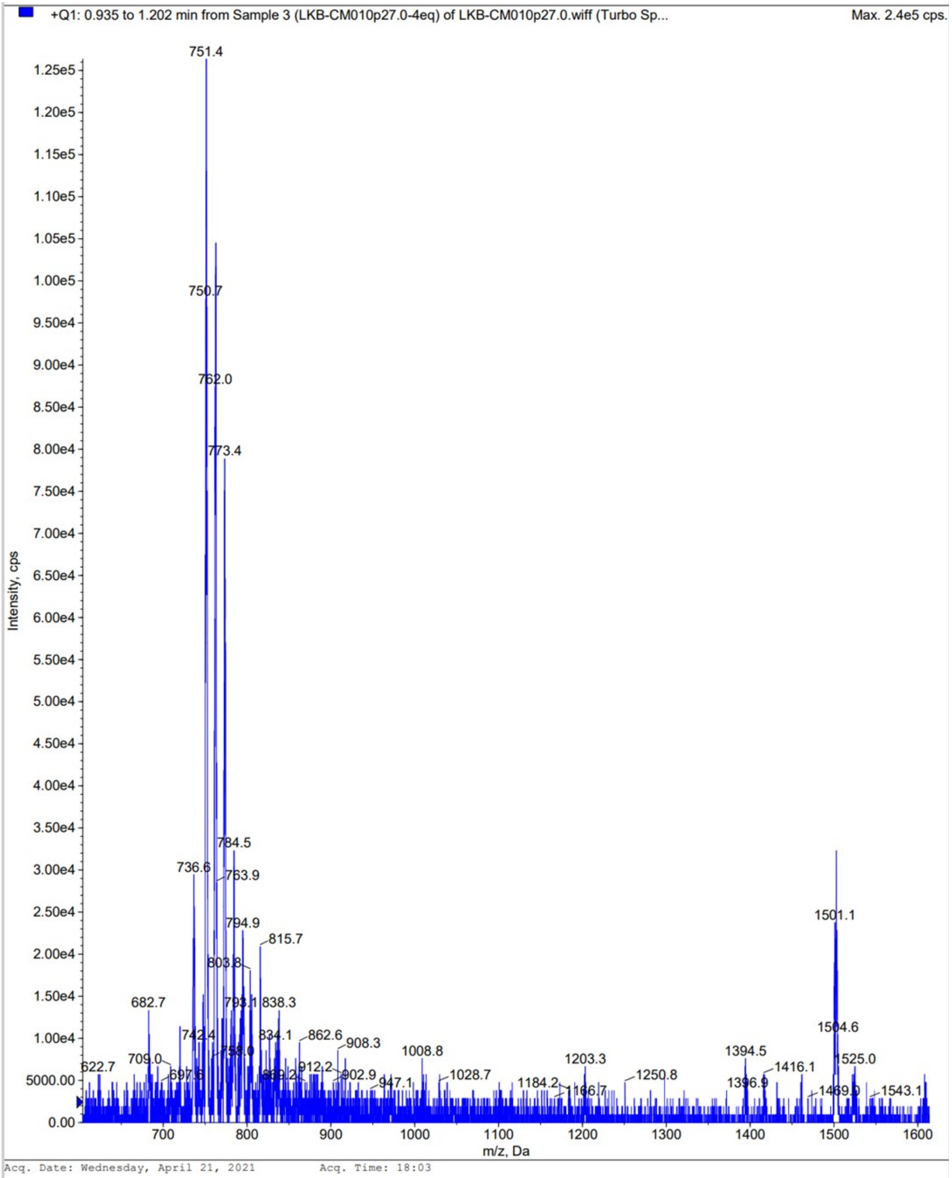

Figure S5. Mass spectra of SilB-p2 in presence of 4 equivalents of silver nitrate at pH 7.0.

Table S1. Mass spectrometry signals of complexes observed when adding 4 equivalents of silver(I) to SilB-p peptides

Peptide	m/z	Intensity
SilB-p1<br><br>(pH 7.0, MW= 1364.6 g/mol)	491.4 $[M+2H+Ag]^{3+}$<br>736.3 $[M+H+Ag]^{2+}$<br>748.3 $[M+Na+H+Ag]^{2+}$<br>758.6 $[M+2Na+Ag]^{2+}$<br>1472.3 $[M+Ag]^{+}$<br>790.6 $[M+2Ag]^{2+}$	9e4<br>4.4e5<br>1.6e5<br>6e4<br>3e4<br>3e4
SilB-p2<br><br>(pH 7.0, MW= 1394.5 g/mol)	751.4 $[M+H+Ag]^{+}$<br>762.0 $[M+Na+Ag]^{2+}$<br>773.4 $[M+2Na+Ag]^{2+}$<br>784.5 $[M+3Na+Ag]^{2+}$<br>1501.1 $[M+Ag]^{+}$<br>803.8 $[M+2Ag]^{2+}$	1.25e5<br>1.25e5<br>9.0e4<br>3.5e4<br>4.0e4<br>2.5e4

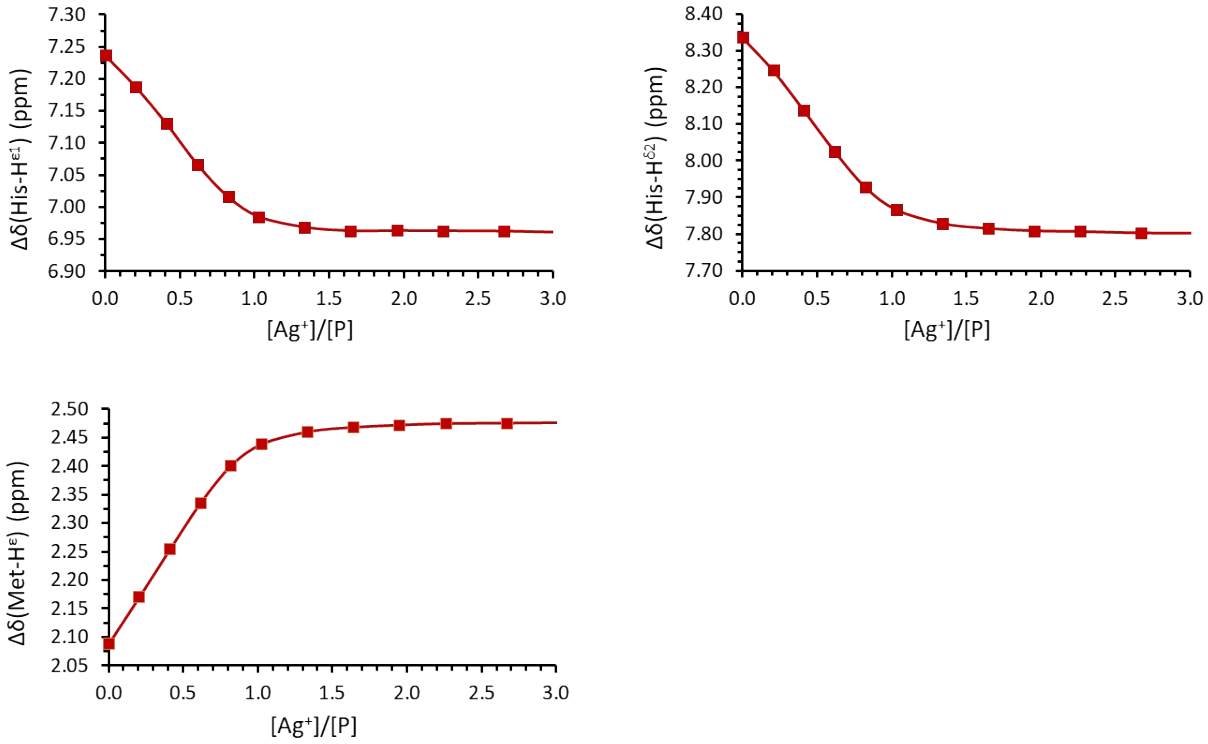

Figure S6. Chemical shift perturbations of the histidine and methionine ^{1}H resonances upon addition of an increasing concentration of $AgNO_3$ (0 to 1.9 mM) to a solution of SilB-p1 (500 μM) in HEPES buffer (20 mM, pD 7.8).

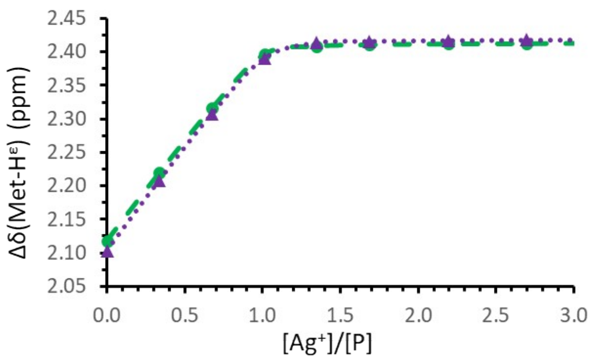

Figure S7. Chemical shift perturbations of the methionines ^{1}H resonances upon addition of an increasing concentration of $AgNO_3$ (0 to 1.4 mM) to a solution of SilB-p2 (300 μM) in HEPES buffer (20 mM, pD 7.8).

Table S2. Overview of the binding constants obtained by ^{1}H NMR spectroscopy in HEPES buffer (20 mM, pD=7.8) for the two different peptides SilB-p1 and SilB-p2. The binding constants were derived from the titration curves obtained in Fig. S6,7 by means of a 1:1 model (see Appendix 1 below).

Peptide	K_d
SilB-p1	8 ± 2 μM
SilB-p2	2 ± 1 μM

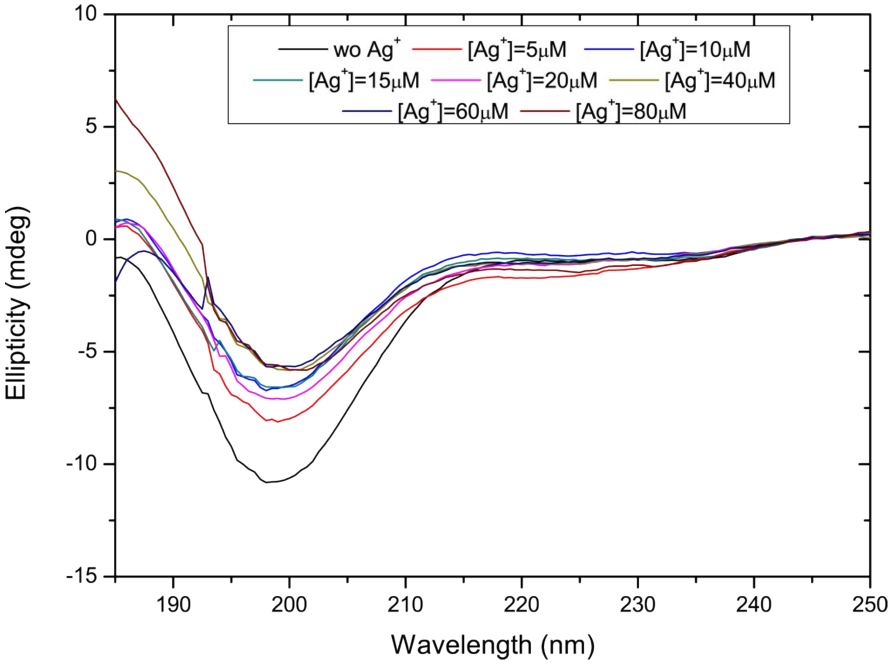

Figure S8. CD experiments during the silver titration of SilB-p (10 µM) in ammonium acetate solution (10 mM, pH = 6.4). Five repetitions have been recorded for each ratio.

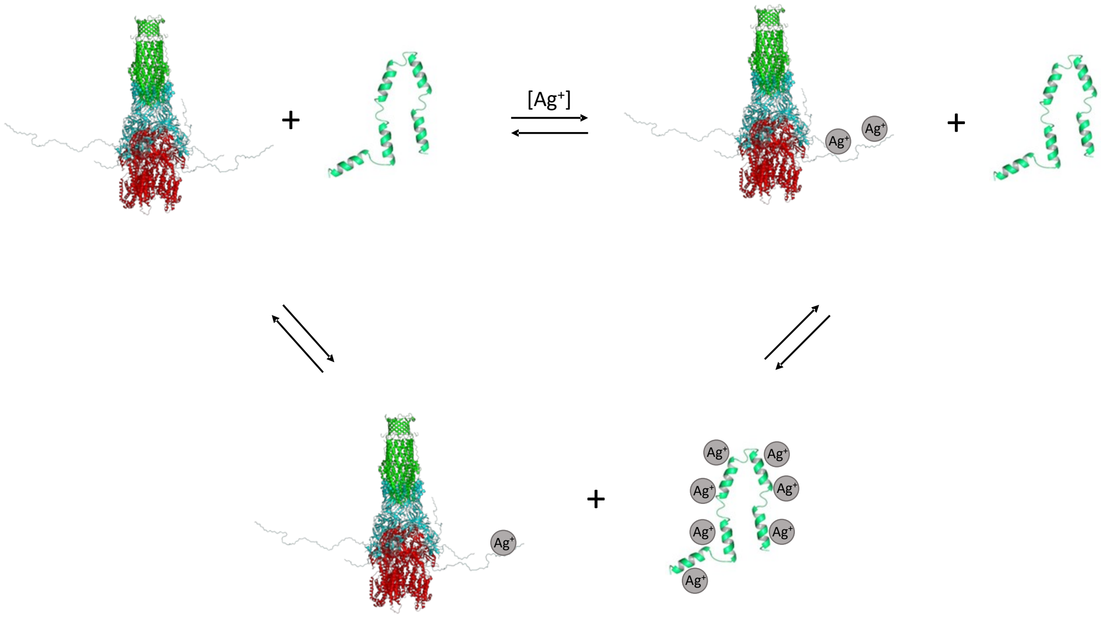

Figure S9. Hypothetical mechanism of the interplay between SilB and SilE derived from our observations. At low silver concentration, the C-terminus of SilB may accommodate two silver ions with a possible further transfer to SilC. When the silver concentration significantly increases, the system triggers a rapid remodeling with SilE acting as a regulator to avoid saturation of the efflux pump.

Appendix 1. Dissociation constants calculation

To derive the corresponding binding constant for the SilE-p/Ag^+ interaction, CSPs were analyzed by calculating the combined amide chemical shift perturbation (Δδ) as $\Delta\delta=[((\Delta\delta_H)^2+(\Delta\delta_N/5)^2)/2]^{1/2}$. By considering a two sites interaction and a 2:1 stoichiometry, we assume that two silver ions can bind to either of the two SilE-p binding sites. The perturbations observed on SilE-p do not discriminate between the two sites so that the observed chemical shift perturbation is a weighted average between the two extreme values corresponding to the free (Δδ=0) and ligand-bound state ($\Delta\delta=\Delta\delta_{LB}$). For a 2:1 binding model, considerations based on partitioning between the free and various ligand-bound states of SilE-p give :

$$\Delta\delta = \Delta\delta_{LB}\left([L_0]+2[P_0]+K_d-\sqrt{([L_0]+2[P_0]+K_d)^2-8[L_0][P_0]}\right)\Big/4[P_0]$$

where $[P_0]$ and $[L_0]$ are the total molar concentrations of SilE-p and Ag^+ respectively.
The dissociation constant K_d and $\Delta\delta_{LB}$ were fitted with non-linear regression by using an in-house Matlab (The MathWorks, Inc) based program. Errors were estimated by sampling 100 initial guesses, assuming 10% error on the protein and ligand concentrations.

To derive the dissociation constant corresponding to the SilB-p/Ag^+ interaction, we have used a two sites sequential model that can be described by:

$$[P]+[L] \underset{k_{-1}}{\overset{k_{+1}}{\rightleftharpoons}} [PL_1]+[L] \underset{k_{-2}}{\overset{k_{+2}}{\rightleftharpoons}} [PL_2]$$

where P stands for the free protein, PL_1 the partly bound protein and PL_2 the totally bound protein. The two dissociation constants can be written as:

$$Kd_1 = \frac{k_{-1}}{k_{+1}} = \frac{[P][L]}{[PL_1]} \quad ; \quad Kd_2 = \frac{k_{-2}}{k_{+2}} = \frac{[PL_1][L]}{[PL_2]}$$

Assuming fast exchange, the chemical shift perturbation can be rewritten as:

$$\Delta\delta = p_P\Delta\delta_P + p_{PL_1}\Delta\delta_{PL_1} + p_{PL_2}\Delta\delta_{PL_2}$$

where p_i is the corresponding populations of the different complexes. The concentrations of the different complexes are:

$$[P]=\frac{[P_T]\cdot Kd_1\cdot Kd_2}{Kd_1\cdot Kd_2+[L]\cdot Kd_2+[L]^2}$$

$$[PL_1]=\frac{[L]\cdot[P_T]\cdot Kd_2}{Kd_1\cdot Kd_2+[L]\cdot Kd_2+[L]^2}$$

$$[PL_2]=\frac{[L]^2\cdot[P_T]}{Kd_1\cdot Kd_2+[L]\cdot Kd_2+[L]^2}$$

where P_T stands for the total protein concentration. Δδ can be recast as:

$$\Delta\delta=\frac{Kd_1\cdot Kd_2\cdot\Delta\delta_P+[L]\cdot Kd_2\cdot\Delta\delta_{PL_1}+[L]^2\cdot\Delta\delta_{PL_2}}{Kd_1\cdot Kd_2+[L]\cdot Kd_2+[L]^2}$$

where the two extreme values correspond to the free ($\Delta\delta_P$=0) and totally bound protein ($\Delta\delta=\Delta\delta_{PL2}$) and [L] is obtained by solving the following cubic equation:

$$[L]^3+[L]^2(Kd_2+[P_T]-[L_T])+[L](Kd_1\cdot Kd_2+Kd_2\cdot[P_T]-Kd_2\cdot[L_T])-[L_T]\cdot Kd_1\cdot Kd_2=0$$

The four parameters comprising K_{d1}, K_{d2}, $\Delta\delta_{PL1}$ and $\Delta\delta_{PL2}$ were fitted with non-linear regression by using an in-house Matlab (The Mathworks, Inc) based program. Errors were estimated by sampling 500 initial guesses, assuming 10% error on protein and ligand concentration.

References

1. Simossis, V. A.; Heringa, J., The PRALINE online server: optimising progressive multiple alignment on the web. *Computational Biology and Chemistry* **2003,** *27* (4), 511-519.

2. Simossis, V. A.; Heringa, J., PRALINE: a multiple sequence alignment toolbox that integrates homology-extended and secondary structure information. *Nucleic Acids Research* **2005,** *33* (suppl_2), W289-W294.
